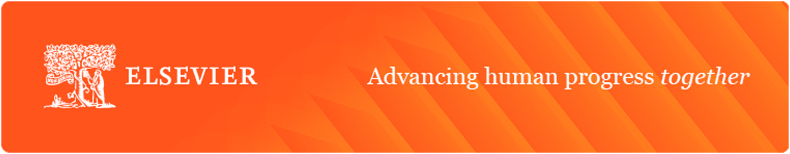# Artificial Intelligence and the future of clinical trials

**DOI:** 10.1016/j.conctc.2025.101545

**Published:** 2025-08-29

**Authors:** Consolato M. Sergi, Howard D. Sesso

**Affiliations:** a Division of Pathology, Children's Hospital of Eastern Ontario, University of Ottawa, Ottawa, ON, Canada; b Department of Laboratory Medicine and Pathology, University of Alberta, Edmonton, AB, Canada; c Division of Preventive Medicine, Brigham and Women's Hospital and Harvard Medical School, Boston, MA, USA; d Department of Epidemiology, Harvard T.H. Chan School of Public Health, Boston, MA, USA

Artificial intelligence (AI), also known worldwide as machine intelligence, is a new approach in the evolution of human beings. It refers to an intelligent approach programmed by humans and exhibited by machines to counter the innate intelligence observed in humans [1]. AI research has grown exponentially in computer science in recent years, and its applications into research and clinical care appear to be an inevitability [2]. Recently, the investigation of “intelligent agents,” which refers to any technology capable of seeing its surroundings and making decisions that optimize its likelihood of accomplishing its objectives, has increased weight in our society. Most conventionally, AI describes a situation where a machine imitates the cognitive abilities that humans typically identify with human minds. It includes “learning modules” and “problem-solving flows”. We assume that AI can replicate a restricted cognitive pattern, enhance the computational capabilities of several tasks, expand the storage capacity in a four-dimensional way, and advance the field of robotics in surgery, medicine, and other fields [[3], [4], [5], [6], [7], [8], [9]].

Clinical trials may be particularly amenable to benefit from advances in AI [[10], [11], [12], [13], [14], [15], [16]]. Clinical trials typically collect large volumes of data with data management and security requirements, as some data may become relevant in the future upon the completion of additional processes. Many groups, including hospitals, academic institutions, and pharmaceutical companies, can enhance their market value by leveraging AI technology, leading to improved effectiveness and efficiency in their research and product development efforts. Personalized treatment modalities determined through AI with big data may have profound time savings and improve quality of life to optimize several diagnostic and therapeutic processes in healthcare and improve patient outcomes and longevity.

## 1 Physicians and AI

Physicians have the potential to enhance the research program in AI by cooperating actively with pharmaceutical companies and those involved with AI in various capacities [17]. AI is currently implemented in the field of drug discovery, including collecting and combining information to develop a comprehensive overview, gain knowledge about the causes and processes of diseases, identify and establish novel and existing biomarkers, generate data and models, and ultimately validate and optimize drug candidates. In turn, AI has also supported the development of pharmaceutical compounds and the formulation and completion of preclinical experiments.

AI has also shown promise in the development of protocols, recruitment, and integration of real-world evidence for the design, conduct, and analysis of randomized clinical trials [7,18,19]. For example, the efficiency of oncology medication development and therapeutical trials has been greatly enhanced by utilizing a biomarker monitoring platform that incorporates a vast amount of patient data points will be critical in this half-century. It is also critical that computer-based simulations of clinical studies be conducted. Cancer bioassays, frequently seen as the gold standard by the National Toxicology Program and other experimental cancer organizations, may soon become obsolete. AI may also inevitably threaten how we typically conduct tests in a controlled environment and within a living organism, which is often laborious and costly. Cost-effective and time-efficient AI algorithms have been successfully utilized to predict the bioactivities of drugs, such as anticancer, antiviral, and antibacterial activities. AI can also be used to examine cancer and other disease-related genetic data. New potential indications for previously approved or candidate medications or supplements may rapidly be clinically validated through a cost-efficient, streamlined process. AI and machine learning can be utilized to identify connections and replicate cognitive processes to assess the efficacy of novel pharmaceuticals or explore alternative therapeutic uses for existing drugs, which will force additional evolution in the field of clinical trials. For example, structure-based virtual screening (SBVS) and ligand-based virtual screening (LBVS) speed up the process of finding potential drug candidates and reduce the number of compounds required for laboratory testing [[20], [21], [22], [23], [24], [25], [26]].

ChatGPT, a widely utilized platform, has over 200 million active users, with one-third of U.S. individuals aged 18 to 64 engaging with generative AI on a weekly basis [27]. The rate of adoption highlights the necessity for systematic, knowledgeable, and purposeful use of AI-informed technologies into clinical research. Prior to the complete integration of technology, a comprehensive evaluation of the benefits and detriments of generative AI in the clinical research sector should be conducted systematically. Risks must be adequately addressed to ensure that the application of generative AI in clinical research is reliable in accordance with ethical, technological, and regulatory standards. In addition to issues about the permissible use of data, meticulous evaluation of training data is essential to prevent the reinforcement of bias. If left unaddressed, such biases might have harmful consequences throughout the clinical research spectrum, affecting the interaction of generative AI with participants, its application in analyzing existing data, and its role in generating new data [28]. Bias in the clinical research process would systemically entrench imbalances in healthcare for future generations and must be avoided at all costs.

## 2 Recommendations and safeguards

In response to various concerns regarding the application of AI in clinical research, regulatory agencies have begun to develop safeguards and guidelines. An Executive Order issued in 2023 on the Safe, Secure, and Trustworthy Development and Use of Artificial Intelligence emphasized the critical importance of oversight in the swiftly evolving domain of generative AI (Executive Order on the Safe, Secure, and Trustworthy Development and Use of Artificial Intelligence WhiteHouse.gov (2023), Accessed 20th Dec 2024). The Center for Drug Evaluation and Research (CDER) has recently formed the CDER AI Council to facilitate coordinated initiatives for regulatory decision-making and enhance support for innovation and best practices in AI-enabled medical products. Regulation is crucial for leveraging the transformative capabilities of generative AI (U.S. Food and Drug Administration Artificial intelligence for drug development https://www.fda.gov/about-fda/center-drug-evaluation-and-research-cder/artificial-intelligence-drug-development, Accessed 17th Dec 2024). The scientific community has made efforts to improve the assessment of AI in healthcare. Guidelines based on international consensus have been established for the development of protocols (Standard Protocol Items: Recommendations for Interventional Trials–Artificial Intelligence) and for the publication of results (Consolidated Standards of Reporting Trials–Artificial Intelligence). These guidelines enhance the evaluation and transparency of methods and results, thereby improving reporting practices in publications [[29], [30], [31], [32], [33], [34], [35], [36], [37], [38], [39], [40], [41], [42], [43], [44], [45], [46], [47]]. The potential of generative AI to erode trust in clinical research is significant, necessitating strategies that promote equity and address biases [48]. Future model assessment must evaluate both the associated risks and the specific use case of the model. Establishing an appropriate level of oversight is essential for regulatory bodies and the clinical research community. Generative AI tools with lower risk, such as those utilized for clinical trial support or document generation, likely require less oversight. In contrast, tools employed in higher-risk scenarios, such as data analysis or clinical trial endpoint adjudication, necessitate significantly stricter scrutiny. Trust and trustworthiness are critical for the integration and adoption of AI in clinical research. Establishing trust necessitates continuous dialogue regarding the capabilities and limitations of AI models, regular assessments, and transparent feedback mechanisms to adapt to shifts in population or medical practices [49]. Establishing strong guardrails and standardized practices across the industry is essential for fostering an ethical AI environment in clinical research. Effective data management practices are essential for ensuring data availability, quality, privacy, security, and for upholding the rigor and reusability of research data. While transparency may differ in early design stages to safeguard intellectual property, essential aspects like training data and model performance should be revealed at final deployment to maintain accountability and trust, all while honoring intellectual property rights.

## 3 The way forward

Establishing industry-wide ethical standards and strong safeguards is essential for the protection of human dignity, privacy, and rights. Regulatory bodies and industry groups should implement compliance enforcement via periodic audits and updates to guidelines. Engagement in forums and discussions among the broader community, including academia and clinical practices, is essential for adapting and refining ethical standards in accordance with technological and societal changes. Foote et al. proposed a deliberate strategy that outlines the successes and failures of AI at each stage of the research process, along with the dissemination of findings and collaboration on improvements [50]. This study proposes a comprehensive mapping of the clinical research process to identify existing bottlenecks and determine where AI could facilitate both minor and major improvements. Dilts and Sandler provided a framework for achieving this step, meticulously documenting the over 700 steps necessary to initiate a phase III oncology clinical trial [51]. Identifying opportunities for the active deployment of generative AI, such as in interactive informed consent, facilitates collaboration among stakeholders. In this context, the generation and presentation of AI-based solutions in an open-access format would accelerate the adoption of generative AI in clinical trials and facilitate the advancement of more transformative technologies. Early demonstration of the benefits and safety of this potential application of generative AI is crucial for building trust among key stakeholders, including trial participants and institutional review boards. It will also show sponsors that generative AI can be effectively integrated into the clinical trial workflow. We strongly reinforce the creation of open-access platforms to improve the integration of AI in clinical research. These platforms facilitate the sharing of training datasets, AI algorithms, and models, thus promoting access to advanced tools and encouraging transparency and collaboration within the field. This may involve the provision of technologies for public access, allowing individuals to use, modify, and distribute them freely. Developers may utilize open-source licenses, leverage platforms such as GitHub or Hugging Face for accessibility, and offer comprehensive documentation and support to assist users. Facilitating the exchange of knowledge and insights among disciplines is essential for promoting innovation in clinical research practices [52].

In conclusion, generative AI in conjunction with digital health is poised to revolutionize clinical research [[53], [54], [55]]. Prior to entrenchment, it is essential to establish regulatory guidelines and safeguards, alongside fostering a culture of continuous monitoring, evaluation, transparency, knowledge sharing, and inclusivity. These initiatives are crucial for ensuring that generative AI advances clinical research and clinical trials in a responsible and equitable way.

**Figure:**